# Cigarette smoking patterns among U.S. military service members before and after separation from the military

**DOI:** 10.1371/journal.pone.0257539

**Published:** 2021-10-04

**Authors:** Chiping Nieh, James D. Mancuso, Teresa M. Powell, Marleen M. Welsh, Gary D. Gackstetter, Tomoko I. Hooper

**Affiliations:** 1 Department of Preventive Medicine and Biostatistics, Uniformed Services University of the Health Sciences, Bethesda, Maryland, United States of America; 2 Leidos, Inc., Reston, Virginia, United States of America; 3 Department of Veterinary and Biomedical Sciences (VBSD), South Dakota State University, Brookings, South Dakota, United States of America; Medical University of South Carolina, UNITED STATES

## Abstract

U.S. military Service members have consistently smoked more than the general population and the prevalence of smoking is even higher among U.S. veterans. Our study examined cigarette smoking patterns among Service members before and after military separation to better understand the disproportionate rate of smoking among veterans. Data from the Millennium Cohort Study were used. All study participants were in the military at baseline and some transitioned from the military to civilian life during the observation period. We investigated any impact of military separation on smoking, as well as other potential risk factors for smoking. Overall, we observed higher smoking prevalence among veterans than Service members. Additionally, we found that Service members smoked more while approaching their separation from the military. Longitudinal analysis revealed military separation was not a risk factor for smoking, as we had hypothesized. Baseline smoking was the most influential predictor of current smoking status. Other significant factors included alcohol consumption, life stressors, and mental health conditions, among others. Military separation was not a risk factor for smoking. However, Service members in the process of transitioning out of the military, as well as high alcohol consumers and Service members with mental health conditions, may be at higher risk of smoking. Including smoking prevention/cessation programs in pre-separation counseling sessions and developing smoking screening and cessation programs targeting specific high-risk subgroups may reduce smoking among Service members and veterans.

## Introduction

Historically, overall tobacco use has been consistently higher among U.S. military personnel compared to civilian populations [1–7]. Tobacco has potentially far-reaching consequences for the U.S. military, including tobacco-related adverse effects on combat readiness, risks to the health and welfare of military beneficiaries, and avoidable costs incurred from added health care services as well as lost productivity. According to the Institute of Medicine, smoking affects military readiness by impairing physical performance and endurance, reducing vigilance and cognitive function, increasing the risk of motor vehicle crashes and other unintentional injuries, as well as resulting in work absenteeism [8]. The health effects of tobacco on all users, including military beneficiaries, include cardiovascular disease, respiratory disease, and cancer, as well as short-term adverse effects, such as acute respiratory illnesses, impaired wound healing, periodontal disease, and peptic ulcer disease [9]. Sadly, tobacco use accounts for one-sixth (nearly 17%) of the deaths among Department of Defense (DoD) beneficiaries, primarily among military retirees [10]. In fact, several investigators have estimated that the cost to the DoD resulting from tobacco use could exceed $1 billion per year [10, 11].

Recognizing the negative impact of smoking on military readiness, the DoD implemented several tobacco prevention/intervention programs, such as tobacco cessation counseling, direct access to specific tobacco cessation products, and instituting new workplace tobacco control policies [12, 13]. Institutional tobacco control policies included creating tobacco-free areas and buildings, restrictions on workplace product promotion and advertisements, and increasing the price for tobacco products within the military commissary system. Although these intervention programs and policies may have contributed to a 21% decline in smoking prevalence between 1980 and 1998, the prevalence of smoking has subsequently increased by 2.3% from 1998 to 2005 [14]. Unfortunately, overseas military deployments were shown to be associated with smoking initiation or resumption [15]. Thus, some of the increases in military smoking trends in the early 2000s may have been reinforced by prolonged deployments to Iraq and Afghanistan.

With efforts to reduce smoking in U.S. military Service members, recent data indicated that smoking prevalence in U.S. military and civilian populations were close to equivalent by 2015, 14% and 15%, respectively [16, 17]. However, smoking prevalence among veterans or those who separated from the military remained as high as 30% from 2010 to 2015 [18]. It is important to note that approximately one-third of veterans who enrolled in the Department of Veterans Affairs health care system never smoked. In contrast, the comparable figure in the U.S. general population was 57% [19]. Military and veteran populations differ from the general U.S. population with respect to age, sex, health status, and socioeconomic status. Hence, tobacco prevention, control and cessation programs used in civilian populations may need to be modified to increase effectiveness among military and veteran communities. While some prospective studies have been conducted to identify smoking behaviors among military and civilian populations, to our knowledge, no studies have examined smoking behavior at the time that active duty military Service members transition to veteran status. Given that this transition period could be stressful to Service members, an analysis of cigarette smoking patterns at this critical time of transition may prove beneficial in identifying more robust tobacco prevention and control measures among both military Service members and veterans. Therefore, the objective of our study was to examine cigarette smoking among military Service members during and after the time of transition in order to better understand and guide targeted smoking cessation, as well as prevention efforts.

## Methods

### Study population

We used data from an ongoing, prospective military study, the Millennium Cohort Study, to examine cigarette smoking patterns before and after military separation. The Millennium Cohort Study population and research methods have been previously described in detail elsewhere [20–22]. In brief, the Millennium Cohort was initiated in 2001 and currently has over 200,000 participants in four enrollment panels, and includes current and former active duty Service members, men and women from the Army, Navy, Air Force, and Marines, as well as National Guard and Reserve components. Panel 1 was drawn from a population-based random sample of the U.S. military in October 2000, with oversampling of Reserve/Guard personnel, women, and Service members with previous deployment experience. Panels 2, 3, and 4 were comprised of new accessions (1–5 years of military service) and oversampled subgroups of Marines and women. Each participant was asked to sign an informed consent before completing an extensive baseline health survey at enrollment. Participants are resurveyed approximately every three years, even after separation from the military. The study was approved by the Naval Health Research Center Institutional Review Board, protocol number NHRC.2000.0007.

**Fig 1:**
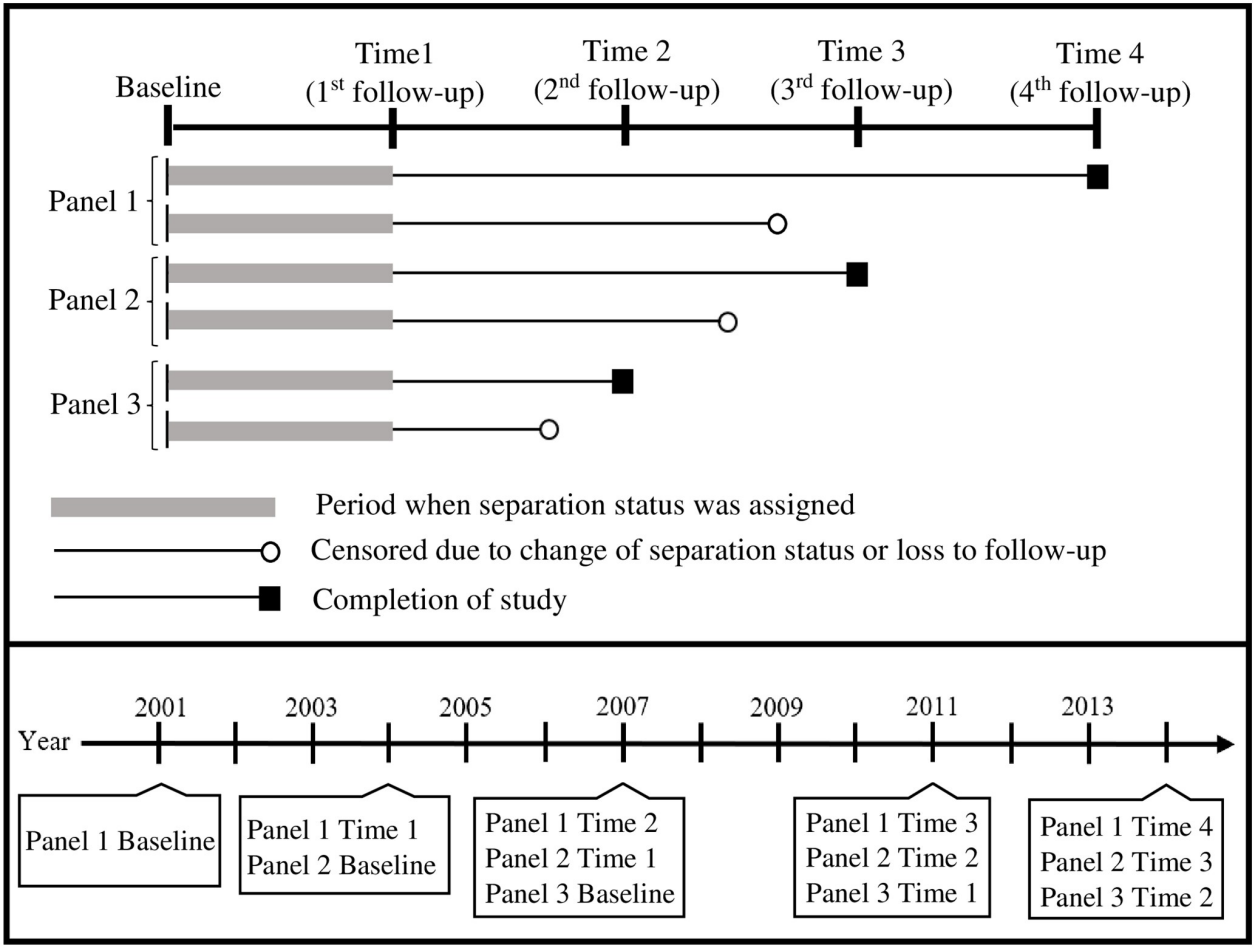
Study design diagram by panel and time point as well as by panel and calendar year.

To examine cigarette smoking associated with transitioning from military to civilian status, we defined our study population as Millennium Cohort Study participants from enrollment panels 1 (enrolled in 2001), 2 (enrolled in 2004), and 3 (enrolled in 2007). Our study population included a total of 151,567 Service members, 77,019 from panel 1, 31,110 from panel 2, and 43,438 from panel 3. We were able to analyze longitudinal data for up to five survey time points: baseline, first follow-up (time 1), second follow-up (time 2), third follow-up (time 3), and forth follow-up (time 4). Follow-up surveys occurred every 3–4 years over a 15-year time period from 2001 to 2016. All study participants were on active duty or in the National Guard/Reserve component at the time of their baseline survey. Military separation status was established at the completion of the first follow-up survey at time 1. Veteran status was defined as those who separated from the military between baseline and time 1. Individuals who changed their separation status after it was assigned, i.e., from veteran back to active duty or vice versa, or who were lost to follow-up were censored (Fig 1). Participants with missing covariate data were eliminated from our analyses.

### Outcome variable

Cigarette smoking, hereafter referred to as smoking, was ascertained at each time point based on participants’ responses to two survey questions, “*In your lifetime*, *have you smoked at least 100 cigarettes (5 packs)*? *(Yes/No*.*)*” and “*Have you ever tried to quit smoking*? *(1*. *Yes*, *and succeeded; 2*. *Yes*, *but not successfully; 3*. *No*.*)*” Participants who answered yes to the lifetime smoking question, and indicated that they had either not tried to quit smoking or had been unsuccessful at quitting were classified as a smoker. Not current smokers were defined as those who answered no to the lifetime smoking question or those who answered yes but had successfully quit smoking.

### Primary exposure variables

In order to increase our understanding of smoking patterns and behaviors in our military study population, as well as any risk factors associated with smoking before and after military separation, we first considered risk factors for smoking at baseline, and then pursued longitudinal analyses of smoking behavior using follow-up survey data. In the baseline smoking analysis, when all study participants were in the military, the primary exposure variable of interest was time remaining in military Service (TRS), measured from the date of completion of the baseline survey to the date of separation, if separated. We then categorized TRS into five groups: 1) less than 6 months, 2) 6 months to less than 1 year, 3) 1 year to less than 2 years, 4) 2 years to less than 3 years, and 5) 3 years or greater. Since follow-up surveys occurred every 3–4 years, participants who had not separated prior to the first follow-up survey (time 1) were included in the 3 years and beyond category. Military separation dates were extracted from a separation file provided by the Defense Manpower Data Center (DMDC).

In our longitudinal analyses, given that the veteran population in our study had separated between baseline and time 1, we used separation status (yes/no) as our primary exposure variable to compare smoking behaviors between veterans and military Service members. Separation status was validated using the separation file described above. For those Service members who separated after baseline, we used personnel pay and roster files provided by DMDC to censor those who rejoined the military after their initial separation.

### Other covariates

Demographic characteristics and military-related variables, including sex, age, race/ethnicity, deployment experience, military rank, Service component, Service branch, and military occupation, were obtained from DMDC. Additionally, education and marital status were obtained from participants’ responses to the Millennium Cohort Study survey. Body mass index (BMI) was calculated from self-reported height and weight. Deployments in support of the operations in Iraq and Afghanistan were first determined based on DMDC deployment records, while possible exposures to combat during deployment were categorized using survey responses to the question of “witnessing or being exposed to a war-related death, physical abuse, dead or decomposing bodies, maimed soldiers/civilians, or prisoners of war or refugees.” Self-reported mental health conditions, including Post-traumatic Stress Disorder (PTSD), depression, and anxiety, were quantified from standardized survey instruments, PTSD Checklist-Civilian Version (PCL-C) and Patient Health Questionnaire (PHQ), embedded within the Millennium Cohort survey instrument [23–25]. Responses to the PCL-C, a standardized 17-item instrument, was used to identify those who with and without PTSD. Participants were classified as PTSD positive if reporting moderate or greater level of at least one intrusion symptom, two hyperarousal symptoms, and three avoidance symptoms, in accordance with the Diagnostic and Statistical Manual of Mental Disorders, 4^th^ edition (DSM-IV). Those with depression were identified based on the 8-item PHQ depression scale if participants endorsed five or more depression items “more than half the days” or “nearly every day” [25]. Anxiety was identified using PHQ and considered present if participants screened positive for either panic syndrome (endorsing all four anxiety attack-related questions) or other anxiety syndrome (experiencing “feeling nervous, anxious, on edge, or worrying a lot about different things” and endorsing at least three of the six anxiety symptoms “more than half the days” in the last month) [24]. Note that all abovementioned measures for mental health conditions were used for screening purposes and not considered diagnostic. Self-reported alcohol consumption, as well as life stressors, were evaluated using a modified version of the Holmes and Rahe Social Readjustment Scale [26, 27], incorporated within the Millennium Cohort survey. Finally, to adjust for possible temporal trends or any potential confounding related to the enrollment panel, we included a variable indicating Panel 1, Panel 2, or Panel 3. Demographic and military-related variables, along with panel, were assessed at baseline. Alcohol consumption, life stressors, PTSD, depression, and anxiety were assessed longitudinally at each survey time point.

### Statistical analysis

Logistic regression was employed in the baseline model to quantify any relationship between baseline smoking status and TRS. Covariates of interest were selected based on the published literature and subject matter expertise, and thus included in the analyses. Unadjusted analyses were performed to explore any crude relationship between smoking status and each covariate of interest. Adjusted analyses were then accomplished by including the primary exposure variable, TRS, and other significant covariates. Linear regressions, along with t-tests, were used to analyze the downward trends of smoking prevalence by time among Service members and veterans.

In our longitudinal analyses, to account for repeated measures within this study population, we used a generalized estimating equations (GEE) approach designed for longitudinal data [28, 29]. An unstructured correlation matrix was assigned with a binomial distribution for current smoking status. The same set of covariates used in the baseline model were also used in our longitudinal analyses, with the addition of baseline smoking status to account for prior smoking history. All analyses were performed using SAS software version 9.4 (SAS Institute Inc., Cary, NC, USA).

## Results

Overall, 151,567 Service members volunteered, consented, completed a baseline survey, and were enrolled in panels 1–3 of the Millennium Cohort. Among these 151,567 Millennium Cohort Study participants, 81,564 Service members met the eligibility criteria (in military service at baseline with completion of the first follow-up survey) and were included in our study. Service members who had missing covariate data (n = 14,535) were then excluded from our analyses, resulting in a final study population of 67,029 participants, among whom 25% (n = 16,433) had separated from the military and were classified as veterans. Given that approximately 18% of eligible participants were excluded from our study due to missing covariate data, we used multiple imputation techniques to ensure that bias was not introduced by using only participants with complete data. These additional analyses resulted in similar findings.

**Fig 2:**
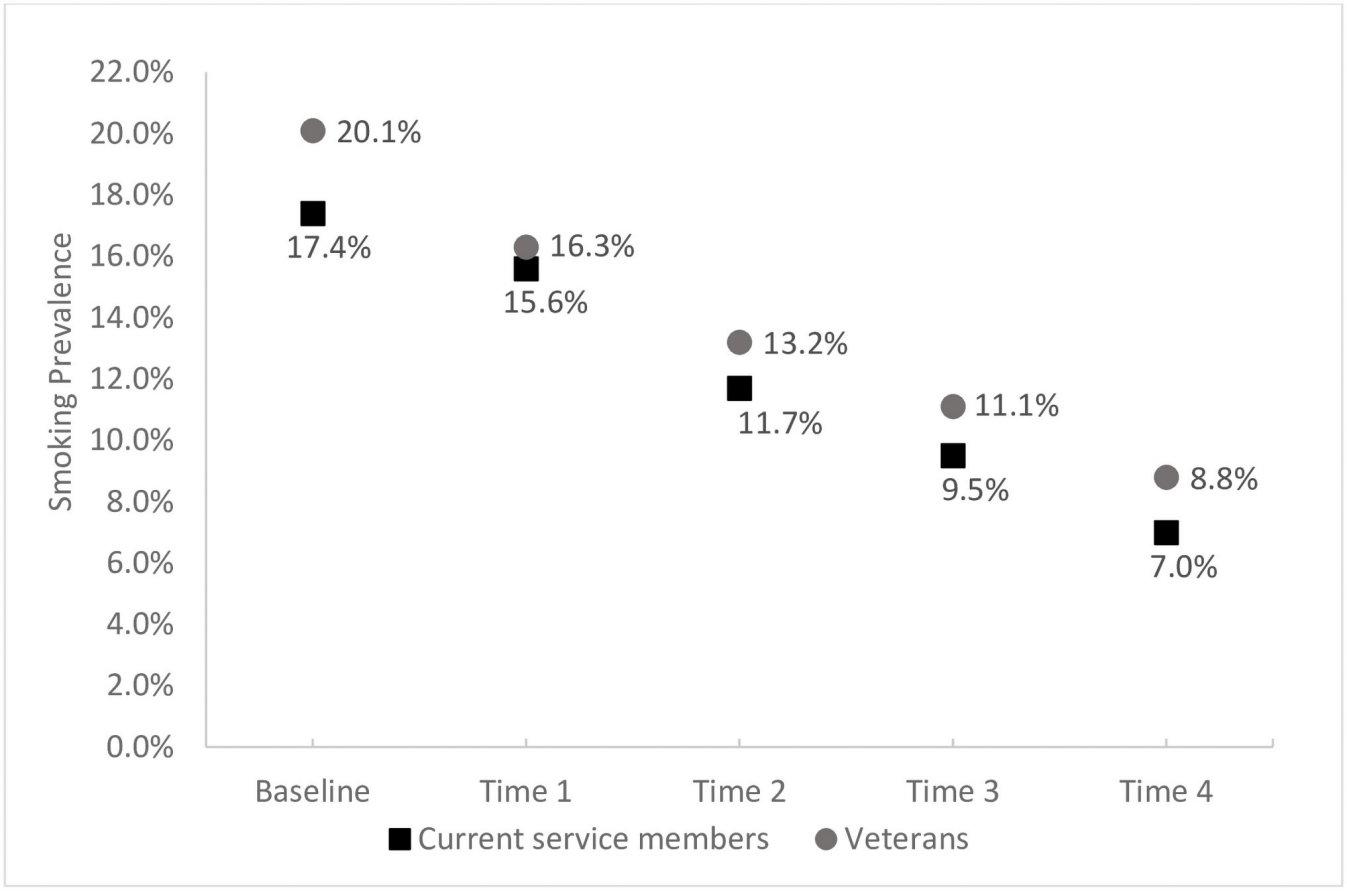
Prevalence of smoking by separation status and time. Both Service members and veterans showed a statistically significant decline in smoking prevalence over time (p = 0.0005 and 0.0009, respectively). Prevalence rates between Service members and veterans are not significantly different at alpha = 0.05 level.

The majority of our study population, shown in Table 1, were men (69.6%), aged 17–34 (66.5%), non-Hispanic whites (76.1%), married (57.3%), and possessed less than a bachelor’s degree from post-secondary education (68.3%). At baseline, compared to our Service member group, our veteran group had higher proportions of younger individuals and were skewed towards being female, not married, less educated, in enlisted ranks, in the Marine Corps, and previously part of the active component. Notably, smoking prevalence at baseline was higher in veterans (20.1%) than in Service members (17.4%). Both Service members and veterans showed a statistically significant decline in smoking prevalence over time (p = 0.0005 and 0.0009, respectively) (Fig 2).

**Table 1:** Baseline demographic and military characteristics, health risk behaviors, and mental health conditions of study participants (N = 67,029), in the Millennium Cohort Study.

Characteristics at baseline	Veterans		Service members		Total	
	No.	%	No.	%	No.	%
Smoking						
Non-smoker	13135	79.9	41813	82.6	54948	82.0
Smoker	3298	20.1	8783	17.4	12081	18.0
Sex						
Male	10767	65.5	35871	70.9	46638	69.6
Female	5666	34.5	14725	29.1	20391	30.4
Age Group						
Age 17–24	6027	36.7	13235	26.2	19262	28.7
Age 25–34	5620	34.2	19730	39.0	25350	37.8
Age 35–44	3006	18.3	13161	26.0	16167	24.1
Age 45+	1780	10.8	4470	8.8	6250	9.3
Race/Ethnicity						
White, non-Hispanic	12623	76.8	38364	75.8	50987	76.1
Black, non-Hispanic	1593	9.7	5733	11.3	7326	10.9
Other	2217	13.5	6499	12.8	8716	13.0
Education						
Less than a bachelor’s degree	11847	72.1	33908	67.0	45755	68.3
Bachelor’s or higher degree	4586	27.9	16688	33.0	21274	31.7
Marital Status						
Single, never married	6113	37.2	15386	30.4	21499	32.1
Married	8581	52.2	29849	59.0	38430	57.3
Separated, divorced, widowed	1739	10.6	5361	10.6	7100	10.6
BMI						
Normal/underweight	7169	43.6	21382	42.3	28551	42.6
Overweight	7463	45.4	24931	49.3	32394	48.3
Obese	1801	11.0	4283	8.5	6084	9.1
Panel						
Panel 1	7165	43.6	30068	59.4	37233	55.5
Panel 2	3376	20.5	8884	17.6	12260	18.3
Panel 3	5892	35.9	11644	23.0	17536	26.2
Rank						
Enlisted	13560	82.5	38142	75.4	51702	77.1
Officers	2873	17.5	12454	24.6	15327	22.9
Service Component						
Reserve/Guard	4282	26.1	20340	40.2	24622	36.7
Active duty	12151	73.9	30256	59.8	42407	63.3
Service Branch						
Army	7812	47.5	22808	45.1	30620	45.7
Navy/Coast Guard	3151	19.2	8494	16.8	11645	17.4
Marine Corps	1802	11.0	2699	5.3	4501	6.7
Air Force	3668	22.3	16595	32.8	20263	30.2
Occupation						
Combat specialist	2750	16.7	9270	18.3	12020	17.9
Health care	2091	12.7	6264	12.4	8355	12.5
Functional support	2922	17.8	9739	19.3	12661	18.9
Electrical/mechanical equipment repair	2329	14.2	7243	14.3	9572	14.3
Other	6341	38.6	18080	35.7	24421	36.4
Deployment						
Not deployed	11166	68.0	39014	77.1	50180	74.9
Deployed without combat exposure	1855	11.3	4847	9.6	6702	10.0
Deployed with combat exposure	3412	20.8	6735	13.3	10147	15.1
Alcohol Consumption						
Abstainer	1422	8.7	3783	7.5	5205	7.8
Infrequent drinker	5579	34.0	16706	33.0	22285	33.2
Light drinker	4134	25.2	13692	27.1	17826	26.6
Moderate drinker	3453	21.0	11951	23.6	15404	23.0
Heavy drinker	1845	11.2	4464	8.8	6309	9.4
Life Stressors						
No stressor	3790	23.1	12470	24.7	16260	24.3
One stressor	6551	39.9	21491	42.5	28042	41.8
Two stressors	3585	21.8	10499	20.8	14084	21.0
Three and more stressors	2507	15.3	6136	12.1	8643	12.9
PTSD						
No	15085	91.8	48665	96.2	63750	95.1
Yes	1348	8.2	1931	3.8	3279	4.9
Depression						
No	15440	94.0	49310	97.5	64750	96.6
Yes	993	6.0	1286	2.5	2279	3.4
Anxiety						
No	15502	94.3	49503	97.8	65005	97.0
Yes	931	5.7	1093	2.2	2024	3.0

When examining factors associated with baseline smoking, TRS was a significant risk factor in the unadjusted model, with all levels of TRS having increased odds of smoking compared to TRS of 3 years or above. We also observed unadjusted dose-response relationships between smoking and age, deployment, and alcohol consumption, as well as number of life stressors (Table 2). Additionally, unadjusted associations between smoking and sex, race/ethnicity, education, marital status, BMI, panel, military rank, Service component, Service branch, military occupation, PTSD, depression, and anxiety were statistically significant at alpha = 0.05 level. In the adjusted analysis, all variables remained statistically significant except for TRS, which was forced into the adjusted model as the primary exposure of interest. The only factor that remained strongly significant in the adjusted model was alcohol consumption (infrequent drinker: adjusted odds ratio [AOR]: 2.29, 95% confidence interval [CI]: 2.04–2.58; light drinker: AOR: 2.90, 95% CI: 2.57–3.26; moderate drinker: AOR: 4.06, 95% CI: 3.61–4.58; heavy drinker: AOR: 6.63, 95% CI: 5.86–7.51). Other factors which retained a modest, but still statistically significant effect in the adjusted model, included life stressors, PTSD, depression, anxiety, and certain demographic and military-related variables (see Table 2).

**Table 2:** Unadjusted and adjusted odds of baseline smoking among study participants (N = 67,029), in the Millennium Cohort Study.

Characteristic	UOR^a^ (95% CI)	AOR^b^ (95% CI)
Time Remaining in Service (TRS)		
Less than six months	**1.20 (1.09, 1.33)**	0.95 (0.86, 1.06)
Six months to less than 1 year	**1.16 (1.06, 1.27)**	0.93 (0.84, 1.03)
1 year to less than 2 years	**1.24 (1.15, 1.32)**	1.02 (0.95, 1.10)
2 years to less than 3 years	**1.16 (1.07, 1.26)**	1.05 (0.96, 1.15)
3 years and beyond	1	1
Sex		
Male	1	1
Female	**0.79 (0.76, 0.83)**	**0.76 (0.72, 0.80)**
Age Group		
Age 17–24	**2.28 (2.10, 2.47)**	1.02 (0.91, 1.13)
Age 25–34	**1.50 (1.38, 1.63)**	1.02 (0.92, 1.12)
Age 35–44	**1.16 (1.06, 1.26)**	**0.90 (0.82, 0.99)**
Age 45+	1	1
Race/Ethnicity		
White, non-Hispanic	1	1
Black, non-Hispanic	**0.55 (0.51, 0.59)**	**0.54 (0.50, 0.58)**
Other	**0.76 (0.71, 0.80)**	**0.71 (0.67, 0.76)**
Education		
Less than a bachelor’s degree	1	1
Bachelor’s or higher degree	**0.20 (0.19, 0.22)**	**0.38 (0.35, 0.42)**
Marital Status		
Married	1	1
Single, never married	**1.36 (1.31, 1.42)**	1.06 (1.00, 1.12)
Separated, divorced, widowed	**1.69 (1.59, 1.79)**	**1.30 (1.22, 1.40)**
BMI		
Normal/underweight	1	1
Overweight	**0.83 (0.80, 0.87)**	**0.79 (0.75, 0.82)**
Obese	**0.82 (0.76, 0.88)**	**0.70 (0.65, 0.76)**
Panel		
Panel 1	1	1
Panel 2	**1.42 (1.35, 1.49)**	**1.08 (1.01, 1.16)**
Panel 3	**1.25 (1.19, 1.31)**	0.99 (0.93, 1.05)
Rank		
Enlisted	1	1
Officers	**0.17 (0.16, 0.18)**	**0.38 (0.35, 0.43)**
Service Component		
Reserve/Guard	1	1
Active duty	**1.31 (1.26, 1.37)**	**1.21 (1.15, 1.28)**
Service Branch		
Army	1	1
Navy/Coast Guard	**0.82 (0.78, 0.87)**	**0.79 (0.74, 0.84)**
Marine Corps	**1.12 (1.04, 1.21)**	**0.74 (0.67, 0.80)**
Air Force	**0.74 (0.71, 0.78)**	**0.71 (0.67, 0.75)**
Occupation		
Combat specialist	**0.87 (0.82, 0.92)**	0.99 (0.93, 1.06)
Health care	**0.65 (0.60, 0.69)**	**0.89 (0.82, 0.96)**
Functional support	**0.85 (0.80, 0.90)**	0.97 (0.91, 1.03)
Electrical/mechanical equipment repair	**1.47 (1.39, 1.56)**	**1.15 (1.08, 1.22)**
Other	1	1
Deployment		
Not deployed	1	1
Deployed, no combat exposure	**1.23 (1.16, 1.32)**	**1.13 (1.05, 1.22)**
Deployed with combat	**1.67 (1.59, 1.76)**	**1.21 (1.14, 1.29)**
Alcohol Consumption		
Abstainer	1	1
Infrequent drinker	**2.39 (2.13, 2.68)**	**2.29 (2.04, 2.58)**
Light drinker	**2.64 (2.35, 2.96)**	**2.90 (2.57, 3.26)**
Moderate drinker	**3.71 (3.31, 4.16)**	**4.06 (3.61, 4.58)**
Heavy drinker	**7.91 (7.02, 8.92)**	**6.63 (5.86, 7.51)**
Life Stressors		
No stressor	1	1
One stressor	**1.15 (1.09, 1.21)**	**1.15 (1.09, 1.22)**
Two stressors	**1.33 (1.25, 1.41)**	**1.27 (1.19, 1.36)**
Three and more stressors	**1.82 (1.71, 1.95)**	**1.69 (1.56, 1.82)**
PTSD		
No	1	1
Yes	**2.26 (2.09, 2.44)**	**1.24 (1.12, 1.37)**
Depression		
No	1	1
Yes	**2.22 (2.03, 2.43)**	**1.25 (1.11, 1.41)**
Anxiety		
No	1	1
Yes	**2.22 (2.02, 2.44)**	**1.25 (1.11, 1.41)**

^a^UOR = Unadjusted Odds Ratio

^b^AOR = Adjusted Odds Ratio

**Bold** numbers are significant at *p* ≤.05

The results of our longitudinal analyses, shown in Table 3, include both unadjusted and adjusted odds ratios for smoking by separation status, as well as selected covariates of interest. Separation was not a risk factor in the unadjusted model. However, when adjusted for baseline smoking and other covariates, separation became a protective factor, and veterans were 20% less likely to be a smoker than Service members. Other variables significantly associated with current smoking status in the unadjusted analysis included smoking at baseline (a 35-fold increase), alcohol consumption, life stressors, PTSD, depression, anxiety, and certain demographic and military-related variables. Notably, deployment without combat exposure appeared to be a protective factor, while deployment with combat exposure was a risk factor. In the adjusted analysis, separation became a statistically significant protective factor for smoking, and the 17–24 age group changed from being a significant risk factor to a protective factor. Baseline smoking remained the most influential risk factor for current smoking status (AOR: 27.32, 95% CI: 25.94–28.78).

**Table 3:** Unadjusted and adjusted odds of current smoking status among study participants (N = 67,029), in the Millennium Cohort Study.

Characteristic	UOR^a^ (95% CI)	AOR^b^ (95% CI)
Separation		
Service members	1	1
Veterans	1.02 (0.97, 1.07)	**0.80 (0.75, 0.85)**
Smoking at Baseline		
No	1	1
Yes	**34.98 (33.32, 36.72)**	**27.32 (25.94, 28.78)**
Sex		
Male	1	1
Female	**0.80 (0.77, 0.84)**	**0.87 (0.81, 0.93)**
Age Group at Baseline		
Age 17–24	**1.96 (1.80, 2.13)**	**0.83 (0.74, 0.92)**
Age 25–34	**1.37 (1.26, 1.49)**	0.92 (0.83, 1.01)
Age 35–44	**1.17 (1.07, 1.28)**	0.95 (0.87, 1.05)
Age 45+	1	1
Race/Ethnicity		
White, non-Hispanic	1	1
Black, non-Hispanic	**0.66 (0.61, 0.71)**	**0.85 (0.78, 0.92)**
Other	**0.76 (0.71, 0.81)**	**0.83 (0.77, 0.90)**
Education		
Less than a bachelor’s degree	1	1
Bachelor’s degree or higher	**0.29 (0.27, 0.30)**	**0.56 (0.52, 0.60)**
Marital Status		
Married	1	1
Single, never married	**1.45 (1.39, 1.51)**	**1.41 (1.32, 1.50)**
Separated, divorced, widowed	**1.49 (1.43, 1.55)**	**1.33 (1.26, 1.42)**
BMI		
Normal/underweight	1	1
Overweight	**0.84 (0.81, 0.87)**	**0.79 (0.75, 0.83)**
Obese	**0.74 (0.70, 0.78)**	**0.63 (0.59, 0.68)**
Panel		
Panel 1	1	1
Panel 2	**1.28 (1.21, 1.35)**	0.93 (0.86, 1.00)
Panel 3	**1.06 (1.01, 1.11)**	**0.84 (0.78, 0.90)**
Rank		
Enlisted	1	1
Officers	**0.20 (0.19, 0.21)**	**0.52 (0.48, 0.57)**
Service Component		
Reserve/Guard	1	1
Active duty	**1.15 (1.11, 1.20)**	**1.07 (1.01, 1.12)**
Service Branch		
Army	1	1
Navy/Coast Guard	**0.82 (0.78, 0.87)**	**0.88 (0.82, 0.95)**
Marine Corps	0.99 (0.91, 1.07)	**0.82 (0.74, 0.92)**
Air Force	**0.66 (0.63, 0.69)**	**0.69 (0.65, 0.73)**
Occupation		
Combat specialist	**0.88 (0.84, 0.92)**	**0.93 (0.87, 0.99)**
Health care	**0.67 (0.63, 0.71)**	0.91 (0.84, 1.00)
Functional support	**0.89 (0.85, 0.93)**	0.97 (0.91, 1.03)
Electrical/mechanical equipment repair	**1.42 (1.35, 1.50)**	**1.13 (1.06, 1.21)**
Other	1	1
Deployment		
Not deployed	1	1
Deployed, no combat exposure	**0.96 (0.93, 0.99)**	**0.85 (0.80, 0.90)**
Deployed with combat	**1.22 (1.17, 1.27)**	1.05 (0.99, 1.11)
Alcohol Consumption		
Abstainer	1	1
Infrequent drinker	**5.95 (4.90, 7.23)**	**2.70 (2.23, 3.27)**
Light drinker	**5.79 (4.77, 7.04)**	**2.78 (2.29, 3.37)**
Moderate drinker	**6.82 (5.61, 8.30)**	**3.25 (2.68, 3.95)**
Heavy drinker	**10.36 (8.49, 12.63)**	**4.25 (3.47, 5.19)**
Life Stressors		
No stressor	1	1
One stressor	**1.10 (1.07, 1.13)**	1.04 (1.00, 1.09)
Two stressors	**1.35 (1.30, 1.41)**	**1.15 (1.07, 1.23)**
Three and more stressors	**1.71 (1.58, 1.84)**	**1.33 (1.20, 1.49)**
PTSD		
No	1	1
Yes	**1.56 (1.47, 1.64)**	**1.17 (1.06, 1.28)**
Depression		
No	1	1
Yes	**1.51 (1.42, 1.61)**	**1.21 (1.09, 1.34)**
Anxiety		
No	1	1
Yes	**1.47 (1.38, 1.56)**	1.01 (0.92, 1.11)

^a^UOR = Unadjusted Odds Ratio

^b^AOR = Adjusted Odds Ratio

**Bold** numbers are significant at *p* ≤.05

Furthermore, the adjusted analysis revealed a strong dose-response relationship between smoking and alcohol consumption (infrequent drinker: AOR: 2.70, 95% CI: 2.23–3.27; light drinker: AOR: 2.78, 95% CI: 2.29–3.37; moderate drinker: AOR: 3.25, 95% CI: 2.68–3.95; heavy drinker: AOR: 4.25, 95% CI: 3.47–5.19), and a more modest dose-response association was found between smoking and life stressors. Smoking was also associated with being non-Hispanic white, Army, on active duty, and screening positive for PTSD or depression. Additionally, the occupational group of electrical/mechanical equipment repair was also associated with smoking. Conversely, participants at reduced risk of current smoking were women, 17–24 years old, who were married, overweight or obese, officers, combat specialists, in panel 3, and college-educated (earned at least a bachelor’s degree).

## Discussion

Within our study population, 20% of those who would soon become veterans and 17% of those who would continue as Service members were identified as current smokers at baseline. Although smoking prevalence declined consistently in both veteran and active duty Service member populations over the 15-year interval of our study, smoking prevalence remained higher among veterans at all time points, confirming the 2009 report by the Institute of Medicine [8]. However, when explicitly examining any association between military separation and smoking, the findings of our study did not fully agree with the hypothesis that the time of transition from the military to civilian life is a risk factor for smoking. We identified, in the unadjusted model, that those who would soon leave active military Service were more likely to smoke at baseline than their counterparts who remained in the military throughout the study time period. However, after adjusting for other covariates, time remaining in Service was no longer a significant risk factor for smoking. In the longitudinal analysis, after adjusting for smoking at baseline and other characteristics, military separation in fact became a protective factor for smoking. We might speculate that serving in the military contributed to a culture of smoking (camaraderie, social norms, peer-pressure) as compared with different experiences at re-entry into civilian life, such as workplace smoking prohibition. It is also possible that Service members lost the financial resources in support of their smoking behavior while on active duty and, therefore, stopped smoking after military separation.

Although we only observed a moderate relationship between TRS and baseline smoking in the unadjusted model, this finding may still suggest that Service members may be smoking more prior to military separation. This ambiguous time period before separation (TRS) is defined by DoD as the time interval starting as early as 2 years before the separation date [30]. Our study findings could prove useful for developing and testing effective smoking interventions to support Service members before and after military separation. Our data did not inform differences among persistent smoking, smoking initiation, and/or smoking relapse, and therefore, we were not able to further explore differences between these groups. Future research focusing on identifying any differences in these groups may prove useful when developing and validating effective smoking prevention/cessation programs tailored to a transitioning population.

We also identified other influential factors associated with smoking in our study. Not surprisingly, smoking at baseline had the strongest association with continued smoking at follow-up. Alcohol use, depression, PTSD, and multiple life stressors also had moderate to strong associations with smoking. The majority of factors significantly associated with baseline smoking and current smoking status in our study were consistent with the existing literature [1, 15, 31, 32]. Baseline and current smokers were more likely to be men, non-Hispanic white, not married, not overweight/obese, in enlisted ranks, and possessing less than a bachelor’s degree from any post-secondary education. Service members in the active component are reported to smoke more than National Guard/Reserve personnel [15], also consistent with our findings.

The finding of deployment being a significant risk factor in our baseline model was aligned with the 2008 study by Smith et al. reporting that Service members smoke to relieve stress during deployment [15]. Interestingly, the effect of deployment on smoking moved towards the null in our longitudal analysis, with deployment without combat experience becoming a significant protective factor. It is possible that since deployment was assessed at baseline, the deployment effect diminished over time. The “healthy warrior effect” could also be a reason for this observation [33, 34]. Since good health status is a prerequisite for military deployment, it is possible that some deployed Service members stopped the smoking behaviors that they developed during deployments and gradually returned back to their healthier lifestyle after deployment.

Our study did identify several risk factors that differed from previous research. When comparing smoking prevalence across military Service branches, we found the highest smoking prevalence in the Army, which differed from DoD reports indicating that the Marine Corps had the highest smoking prevalence, followed by the Army [1, 3, 16]. It is possible that the lower smoking prevalence we observed in Marine Corps members was due to volunteer bias [35] since the Millennium Cohort participation rate for Marines was only 13%, compared to 26% in all other Services combined. It is also possible that health-oriented Marines may have been more willing to respond to a health-focused survey, thereby influencing the smoking rates found in our study.

### Limitations

Our study has several limitations. Smoking status, as well as other variables, were self-reported and, thus, subject to misclassification. Those who volunteered to participate in the Millennium Cohort Study may have been less likely to be current smokers at baseline due to the healthy volunteer effect [35], as suggested by the lower prevalence of smoking in our study population compared to previous studies of military populations. Additionally, in order to examine Service members’ smoking status during the specific period of time of separting from the military, we categorized our participants as current smokers and not current smokers. Those who previously smoked but successfully quit were grouped with the never smokers as not current smokers, which could be subject to misclassification. However, knowing the potential psychosocial differences between never smokers and former smokers, we included former smoking status in all our models to minimize any potential bias that could be introduced due to our grouping method. Futhermore, our study was completed before E-cigarettes gained popularity among smokers and younger initiators, and therefore, we were not able to examine how the use of these new nicotine-delivery devices may be impacting traditional smoking behaviors among both Service members and veterans. Finally, our veteran population is younger than the Service members in our study which implies that we captured a veteran population of those who served a few tours rather than those who built a career in the miltiary before separation. Therefore, our study population is not representative of the veterans who had a full military career.

### Strengths

Despite these limitations, our study had important strengths, including a substantial sample size, a prospective design, and the ability to assess alcohol consumption, life stressors, and multiple mental health conditions, as well as combat experiences (not usually captured in medical encounter data). The large sample size allowed us to control for multiple potential confounders while maintaining adequate statistical power. The longitudinal study design and use of self-reported data enabled us to follow participants throughout the transition process from active duty military service to years after their separation from the military, which is not possible on this scale using current electronic administrative data and medical records.

## Conclusions

In summary, military separation was not a risk factor for smoking, as we had hypothesized. Even though we found that Service members who were close to separation from the military smoked more than those whose separation was further into the future, this association did not retain statistical significance after adjusting for confounding factors. Our study did show that the prevalence of smoking declined among both veterans and currently serving military members over our defined follow-up period. Not surprisingly, baseline smoking was a strong predictor of smoking status at follow-up, suggesting that senior leaders should continue to actively support and strengthen smoking prevention and cessation efforts across the continuum of military service, starting at accession. Additionally, our study indicates that Service members transitioning out of the military may be at increased risk of smoking prior to their actual separation date. We also observed a strong dose-response relationship between smoking and alcohol consumption, as well as a modest association between smoking and life stressors, supporting the use of smoking cessation and prevention programs targeting specific groups. While confounding factors may have played a role in precisely quantifying these associations, increasing the emphasis on smoking cessation and prevention programs in any pre-separation counseling sessions may still reduce the number of smokers among these higher prevalence populations, and that is a worthy public health goal.
